# Relationships of irrigation water and soil physical and chemical characteristics with yield, chemical composition and antimicrobial activity of Damask rose essential oil

**DOI:** 10.1371/journal.pone.0249363

**Published:** 2021-04-16

**Authors:** Mansureh Ghavam

**Affiliations:** Department of Range and Watershed Management, Faculty of Natural Resources and Earth Sciences, University of Kashan, Kashan, Iran; University of the Witwatersrand, SOUTH AFRICA

## Abstract

Damask rose (*Rosa damascena* Mill.) is an aromatic medicinal plant rich in bioactive compounds with high value in the food, pharmaceutical and cosmetic industries. Knowledge of the factors affecting the quantitative and qualitative properties of the compounds in its essential oil (EO) and the bioactivity of this EO is important in optimizing Damask rose cultivation and production. This research studied, for the first time, the effects of irrigation water and soil chemical and physical characteristics on the EO yield of this important commercial species and on it chemical composition and antimicrobial activity. The results showed the significant effect of crop cultivation site on yield, chemical composition and inhibition zone diameter (IZD) at the 1% significance level. The highest EO yield (~0.0266%), which belonged to the Noushabad site (EO_N_), resulted from the increased soil electrical conductivity (EC) and the higher sand, gypsum and lime contents and irrigation water salinity. Analysis of the chemical composition of the EOs showed that their main compounds at all three crop sites were citronellol, nonadecane, heneicosane and geraniol. The EO obtained from the Yazdel site (EO_Y_) had the highest contents of citronellol and geraniol (~29.05% and ~6.85%) that were directly correlated with soil potassium and phosphorus contents and inversely correlated with soil acidity and EC and its lime, nitrogen, and organic carbon contents. Antimicrobial assays indicated that the EO extracted from the Sefidshahr site (EO_S_), which had the largest inhibition zone diameter (~14.67 mm) for *Aspergillus brasiliensis* (IZD~14.67 mm) and the lowest MIC (~31.25 μg/mL) for *Staphylococcus aureus* and *Pseudomonas aeruginosa*, exhibited efficacy similar to that of rifampin, probably due to the dominance of the alkanes in it. The EO_Y_ and EO_S_ also exhibited the strongest inhibitory and lethal activity against *Candida albicans* (MIC and MBC <15.63 μg/mL for EO_Y_ and MIC and MBC = 62.5 μg/mL for EO_S_), which were six and four times stronger than those of nystatin, respectively. Therefore, the selected EOs can act as a potentially promising strategy for fighting microbial strains.

## 1. Introduction

Essential oils (EOs) are the complex content of volatile organic compounds, which are synthesized in aromatic vascular plants as a defense mechanism for antifungal, antiparasitic, antiviral and antibacterial activities. Likewise, they can perform similar activities in the human body [1, 2]. They have been screened and used in pharmacology, medical microbiology and phytopathology [3] and they are considered an available source of chemical diversity to be used for a wide range of infectious diseases even against chemical-resistant strains [4, 5].

Damask rose (*Rosa damascena* Mill.) is one of the most important species in the Rosaceae family [6] with high value products, and its essential oil (EO) is one of the most expensive EOs in the world markets [7, 8]. Apart from its use as an ornamental plant in parks, gardens, and houses, *R*. *damascena* is principally cultivated for the perfume, pharmaceutical and cosmetic industries [9].

In traditional Iranian medicine, more than a thousand years ago Avicenna (980–1037 AD) explained the therapeutic effects of Damask rose such gastrointestinal and cardiac tonic effects, elimination of the unpleasant body odor related to sweat, skin repair and healing of mucosal lesions [10]. Later, Aghili Shirazi in the 12^th^ century AH (1688–1785 AD) described the therapeutic effects of *R*. *damascena* in his book entitled “Medicine Repository” and referred to the plant as a brain tonic and analgesic for various diseases [11]. The most important healing effects of Damask rose EO include reduction of depression, sadness, stress, and thirst, as well as wound healing and skin health improvement. Studies have shown that vapor therapy using this EO may be helpful in treating some allergies, headaches and migraines [12, 13].

Results of clinical trials have shown that *R*. *damascena* EO has tranquilizing effects without any serious complications [14–16]. Various reports also demonstrated its biological activities including antioxidant [17, 18], anti-cancer [19], anti-inflammatory [20], anti-HIV [21, 22], antifungal, antibacterial, and antimicrobial [16, 23–25] properties.

Alkanes, alcohols, phenols, terpenes and terpenoids are the compounds found in Damask rose EO. Its most important alkanes are nonadecane, eicosane, heneicosane, heptadecane and octadecane [26–28] and its major terpene and terpenoid compounds citronellol, geraniol, neral, linalool, and farnesol [29]. Citronellol, geraniol and neral are the main constituents of Damask rose EO contributing to its quality [30], have wide applications in the perfume, cosmetic and soap industries and exhibit potent antimicrobial activity against some bacterial [31].

This plant has been cultivated in many countries including Iran, the United States, the United Kingdom, Bulgaria, Turkey, Japan, and India. It grows wild in many parts of the world and is widely distributed in North America, Europe, Asia and the Middle East [7, 32]. *R*. *damascena* has been cultivated for various purposes in Iran from ancient times, and this country has a long history of producing and exporting its EO around the world [33, 34].

Knowledge of the factors influencing its EO yield and chemical composition is very important for producers of Damask rose [35, 36] Like the other aromatic plants, its EO is affected by genetic and many environmental factors including crop location characteristics [26, 37]. Studies have shown that environmental factors (e.g., annual rainfall, temperature, humidity, light, soil, pruning, provision of nutrients and harvest time) considerably influence Damask rose EO [38–43]. Soil and irrigation water characteristics are among the most important factors influencing the EOs of plants in a field. Soil characteristics are effective factors in growth and development and quantitative and qualitative yields of plants [44]. The chemical elements in the rhizosphere (such as contents of mobile phosphorus and mobile potassium) enter into the composition of the enzymes that take part in the biochemical reactions in plants. Therefore, soil chemistry can affect the composition of essential oils (such as linalool, citronellol, geraniol, eugenol, etc) and also the distribution of chemotypes [45]. Water is also one of the very important environmental factors that affect growth, quality and quantity of the EOs of cultivated medicinal plants. It is not yet clearly known why plants produce EOs; however, EOs are generally the remaining products of the major metabolic processes in plants, especially under stress conditions [46, 47]. In plants that produce EOs (complex mixtures of volatile compounds generally produced in the biosynthetic pathways of terpenoids or phenylpropanoids), interactions with the environment usually lead to changes in the compounds found in the EOs and in their contents [48]. These changes may contribute to the evolution of various plant genes that favor adaptation to the environmental conditions at crop sites thus leading to changes in the quantity and quality of the EOs (and hence in their biological activities) [49, 50]. Therefore, evaluation of genotypes collected from cultivation locations having different environmental conditions is an important step in Damask rose breeding programs prior to selection of desirable cultivars for commercial production.

The effects of irrigation water and soil characteristics on Damask rose EO have not been studied for a specific climate region in Iran. Therefore, this research aimed to: a) study and compare the yield, chemical composition and antimicrobial activity of *R*. *damascena* EO under diverse soil and irrigation water characteristics, and b) select an ideal crop site with optimal soil and irrigation water characteristics to produce the best essential oil with respect to quantity, quality and antimicrobial activity.

## 2. Materials and methods

### 2.1. Selection of cultivation area and sites

The various areas where the Damask rose species of interest was planted in Kashan were identified through field studies to select the sampling area. The Kashan plain was selected since the crop sites in it, which were at suitable distances from each other, had similar climatic and topographical conditions and different soil and irrigation water characteristics. The three selected crop sites of Sefidshahr, Yazdel and Noushabad were 15 km apart. They differed in soil and irrigation water characteristics but had identical planting, growing and harvesting conditions. The geographical characteristics of the studied sites and the climatic features of the area are shown in Table 1.

**Table 1:** Geographical characteristics of the studied crop sites in the Kashan plain.

Site	Longitude	Latitude	Altitude (m)
Sefidshahr	N 37˚ 71.9ʹ 07ʺ	E 53˚ 64.2ʹ 39ʺ	870
Yazdel	N 37˚ 70.3ʹ 75ʺ	E 53˚ 34ʹ 34ʺ	885
Noushabad	N 37˚ 77.8ʹ 08ʺ	E 53˚ 88ʹ 43ʺ	900

### 2.2. Plant material sampling

When Damask rose flower buds began to open in May 2019, flowers (petals and sepals) were randomly collected from different bushes (100 bushes at each crop site) at 6 a.m. The samples were transferred to the laboratory and kept for an hour at 4°C. A complete bush was also collected from each field, identified at the herbarium of the School of Natural Resources and Earth Sciences in Kashan University, coded and stored.

### 2.3. Soil sample collection

At each crop site, soil samples were randomly collected from three points at the depth of 30 cm prior to planting. The samples were transferred to the soil laboratory to determine their physical and chemical characteristics. They were passed through a 2-mm sieve and then prepared for the various tests.

### 2.4. Irrigation water sample collection

Irrigation water samples were collected at each site and transferred to the laboratory to assess water quality characteristics including EC (Electrical Conductivity), acidity, hardness and anions and cations contents. The characteristics of the irrigation water at the three sites are presented in detail in Table 2.

**Table 2:** Irrigation water characteristics at the studied crop sites.

Water characteristics	Site	content
EC (ds/m)	Sefidshahr	2.02
	Yazdel	2.23
	Noushabad	5.49
pH	Sefidshahr	7.236
	Yazdel	7.07
	Noushabad	6.81
TDS (mg/L)	Sefidshahr	1292.80
	Yazdel	1427.00
	Noushabad	3513.00
CO_3_^2-^ (meq/L)	Sefidshahr	0.00
	Yazdel	0.00
	Noushabad	0.00
HCO_3_^-^(meq/L)	Sefidshahr	2.45
	Yazdel	5.40
	Noushabad	3.90
CL^-^(meq/L)	Sefidshahr	8.45
	Yazdel	8.51
	Noushabad	31.41
SO_4_^2-^(meq/L)	Sefidshahr	5.54
	Yazdel	6.99
	Noushabad	19.55
Ca^2+^(meq/L)	Sefidshahr	4.30
	Yazdel	6.76
	Noushabad	14.88
Mg^2+^(meq/L)	Sefidshahr	4.20
	Yazdel	3.82
	Noushabad	11.04
Na^+^(meq/L)	Sefidshahr	4.94
	Yazdel	10.34
	Noushabad	28.91
S.A.R (mmolL) ^0.5^	Sefidshahr	3.85
	Yazdel	4.49
	Noushabad	8.03
S.S.P (%)	Sefidshahr	48.30
	Yazdel	49.42
	Noushabad	52.73
T.H (mg/L)	Sefidshahr	425.00
	Yazdel	529.20
	Noushabad	1282.50
R.S.C(meq/L)	Sefidshahr	-6.05
	Yazdel	0.00
	Noushabad	0.00

### 2.5. Laboratory operations

#### 2.5.1. EO extraction and separation

To extract the EO, fresh flowers (350 g) were collected from each harvesting point at each crop site, weighed and put into a two-liter flask. The EO was extracted by distilled water using a Clevenger-type apparatus for 5 h. The extract was dehydrated using sodium sulfate, separated, and kept in the dark in a dark-colored bottle at 4°C until later use. The EO yield for each harvesting point was expressed based on weight/weight percent (% w/w) at each site. The EO yield for each site was reported as the mean± SD of three replications (harvesting points).

#### 2.5.2. Identification of the compounds in the EOs using GC-MS

A GC-MS instrument was employed to determine the chemical composition of the EO samples. The instrument consisted of a 6890 gas chromatograph (GC) coupled to an Agilent 5973 N mass spectrometer (MS) with a capillary column of HP-5 MS and 5% methyl phenyl of the stagnant phase (30 m length, 0.25 mm internal diameter, the thickness of stagnant layer 0.25 μm) and the ionization energy of 70 eV.

The oven temperature program for the analysis was as follows: it was set at 60°C and raised by 3°C per minute to reach 246°C. The injector and the detector temperatures were set at 250°C. The injection volume of sample was 1μL, split mode (1.50). The helium carrier gas flow was 1.5 mL/min.

The chemical constituents of the EOs were determined based on the GC-MC analysis of each EO sample in relation to the retention indices (RI) and standard mixtures of n-alkane (C8-C20) mixtures and mass spectral data of each peak using spectral libraries (NIST-14 and Wiley-14) and comparing the obtained results with those in the literature [51].

#### 2.5.3. Determination of antimicrobial activity

*2*.*5*.*3*.*1*. *Microbial strains*. Eleven standard microbial strains were used to assess the antimicrobial activities of the EOs. These strains were four Gram-positive bacteria (*Staphylococcus epidermidis* CIP 81.55, *S*. *aureus* ATCC 29737, *Streptococcus pyogenes* ATCC 19615, and *Bacillus subtilis* ATCC 6633), five Gram-negative bacteria (*Klebsiella pneumoniae* ATCC 10031, *Escherichia coli* ATCC 10536, *Pseudomonas aeruginosa* ATCC 27853, *Salmonella paratyphi A* serotype ATCC 5702, and *Shigella dysenteriae* PTCC 1188, and the two fungal strains of *Candida albicans* ATCC 10231 and *Aspergillus brasiliensis* ATCC 16404. The microorganisms were obtained from the Iranian Research Organization for Science and Technology (IROST). The bacterial strains were cultured on nutrient agar and the fungal strains on Sabouraud dextrose agar (SDA). The plates inoculated with the bacteria and fungi cultures were incubated overnight at 37°C and 30°C, respectively.

*2*.*5*.*3*.*2*. *Agar well-diffusion method*. The protocol of the Clinical and Laboratory Standards Institute [52] was employed in using this method. Plates containing Mueller-Hinton agar and SDA were first prepared to culture the bacterial and fungal strains, respectively. The EO was dissolved in dimethyl sulfoxide (DMSO) and the concentration was raised to 300 μg/mL. The turbidity of each microbial suspension (100 μL) was adjusted to that of a 0.5 McFarland standard and the suspensions were cultured on the culture media under identical conditions. A number of wells with 6 mm diameter and 4 mm thickness were made in the culture plates and 10 μL EO (at concentration 300μg/mL) was added to each well. The plates were kept at 4°C for 2 h. The plates inoculated with the bacterial strains were then incubated at 37°C for 24 h and those incubated with the *A*. *brasiliensis* ATCC 16404 and *C*. *albicans* ATCC 10231 were incubated at 30°C for 72 and 48 hours, respectively. Gentamicin (10 μg/disc) and rifampin (5μg/disc) were used as positive controls for the bacterial strains and nystatin (100,000 units/mL) as the positive control for the fungal strains under the same conditions as those for the EO tests. IZD was measured using a ruler (that measures in millimeters) to generate the antibiogram. The test was performed in triplicate for each EO to assess reproducibility and become certain of the reliability of the results. The IZDs were reported as mean ± SD.

*2*.*5*.*3*.*3*. *MIC*. To determine the MICs for the bacterial and yeast strains, sterile 96-well microtiter plates and the broth microdilution method were used according to the CLSI protocol [52]. Various dilutions of the EO were prepared first. A certain volume of the EO was weighed, dissolved in the culture medium and DMSO at a suitable ratio to prepare the initial stock at a concentration of 2000 μg/mL. This stock was used to prepare the 1000, 500, 250, 125, 62.5, 31.25, and 15.63 μg/mL dilutions. Each well in the microplate was filled with 200 μL of a solution containing 95 μL of brain heart infusion (BHI) broth for bacteria or with 95 μL of SD broth for yeast, and 5 μL of the microbial suspension with the turbidity adjusted to that of a 0.5 McFarland standard and 100 μL of one of the EO dilutions were added. The wells intended as negative controls were filled with the culture medium instead of the essential oil and for the wells intended as positive controls antibiotic powders (gentamicin and rifampin) were used instead of the EO for the bacterial strains and nystatin powder for the yeast. Plates inoculated with bacterial and fungal plates were incubated at 37°C for 24 h and at 30°C for 48 h, respectively. MIC was determined taking into account the turbidity and the change in the color in each well of the microplate. The test was performed in triplicate for each EO sample and the mean was reported as the minimum concentration of the EO that inhibited the growth of bacteria or yeast.

The agar dilution assay was used to determine the MICs for the fungal strains based on the protocol introduced by of [53]. The suitable amounts of EO at different concentrations (2000, 1000, 500, 250, 125, 62.5, 31.25 and 15.63 μg/mL) were added to SDA containing 50% (v/v) Tween 20. Nystatin powder was used as the positive control, and the negative control was the plate with SDA containing 50% (v/v) Tween 20 without any EO. The culture media were spot inoculated with 4 ml of spores (10^4^ spores /mL). The inoculated plates were incubated at 30°C for 72 h, the test was performed in triplicate for each essential oil, and the minimal concentration of the essential oil that inhibited the growth of the fungi was reported as the MIC.

*2*.*5*.*3*.*4*. *MBC*. The broth microdilution method based on CLSI protocol (2012) was used as described above to determine MBC. Following 24 h of incubation, the nutrient agar medium was inoculated with 5 μL of each well that exhibited no trace of bacterial growth (light well) and incubated at 37°C for 24 h. MBC refers to the minimal concentration of the EO that kills 99.9% of the inoculated bacteria.

#### 2.5.4. Soil characteristics

Soil pH, EC and phosphorous, potassium, organic carbon, nitrogen, lime and gypsum contents and its texture components were measured using a pH meter, an EC meter, the Olson method, the ammonium acetate extraction method, the Walkley and Black method (1934), the Kjeldahl method, the acid-base titration method, the acetone method, and the hydrometric method, respectively (Zargoosh et al., 2019).

### 2.6. Statistical analysis

SPSS was used for statistical analysis. Data normality was checked using the Kolmogorov-Smirnov test. When normality of the data was proved, one-way and univariate ANOVA were employed to determine significance of differences. Duncan’ test at the 1% significance level was used for comparison of the means. All data were expressed as mean ± SD, Pearson correlation was used to study the correlations between EO yield and the dominant compounds in EO and soil chemical characteristics.

## 3. Results

### 3.1. EO yield

*R*. *damascena* EO was pale yellow at all three crop sites. ANOVA results regarding EO yield showed the significant effect of crop site on EO yield at the 1% significance level (P≤ 0.01) (Table 3). These results are consistent with those of the study by [54]) that the effect of location on yield of *Satureja khuzestanica* Jamzad and *Satureja rechingeri* Jamzad was significant (p< 0.01). Comparison of the means at the three crop sites (Table 4) showed that EO_N_ and EO_Y_ with the mean yields of ~ 0.00266% and ~ 0.0157% had the highest and lowest EO yields, respectively. These results are consistent with those reported in the research by [55] in the Western Himalayas and by [38] in Iran on this plant. However, the highest EO yield of Damask rose in Iran (0.20%) was obtained in Gilan Province [56]. Many studies have assessed the plant-soil interaction effects and have shown the correlation between plant and soil characteristics [57–60]. The present study also investigated the correlations between plant and soil characteristics.

**Table 3:** ANOVA of the effect of crop site on yield and some dominant and important compounds of *R*. *damascena* EO.

Source of variation	df			MS				
		EO yield	Citronellol	Geranial	Nonadecane	Heneicosane	Eugenol	Methyleugenol
Site	2	0.000091**	150.765**	30.942**	107.360**	37.015**	2.043**	2.524**
Error	6	0.0000	0.000	0.001	0.000	0.000	0.000	0.000

**Table 4:** Comparison of the means related yield of *R*. *damascena* EO at the studied crop sites.

Site	Mean (%) ± SD
Sefidshahr	0.0003^b^ ± 0.0224
Yazdel	0.0006^c^ ± 0.0157
Noushabad	0.0001^a^ ± 0.0266

The different letters indicate a significant difference based on Duncan’s multiple range test at the 1% level.

Table 5 shows the results related to correlations between soil characteristics and EO yield. There were direct correlations between EO yield and soil EC and percentages of its sand, gypsum and lime contents and inverse correlations between EO yield and soil silt and clay percentages. The highest values of EC (~4.817 ds/m), sand percentage (~82.000%) and gypsum content (~0.703%) belonged to EO_N_ and the highest values of clay percentage (~11.667%) and silt percentage (~18.67%) were those of EO_Y_ (Table 6). Therefore, lighter soil texture, higher EC (salinity), and higher gypsum and lime contents probably increased EO yield. The plants growing in soils with higher clay percentage must spend more energy for root growth and water and nutrient absorption and hence plant growth and yield decrease. [54] also showed lower EO yield in soils with high clay percentages. Their results are consistent with those of the present study.

**Table 5:** Correlations between soil characteristics and yield and some dominant and important compounds in *R*. *damascena* EO.

Correlation	Silt	Clay	Sand	Gypsum	Lime	N	O.C	K	P	EC	pH
Citronellol	0.266^ns^	-0.259 ^ns^	-0.148^ns^	-0.439^ns^	-0.831**	-0.983**	-0.994**	0.969**	0.894**	0.138 ^ns^	-0. 746*
Geraniol	0.874 **	0.512 ^ns^	-0.812**	-0.423^ns^	-0.943**	-0.483 ^ns^	-0.540 ^ns^	0.418 ^ns^	0.910 **	-0. 684*	0.049 ^ns^
Nonadecane	-0.347^ns^	0.179 ^ns^	0.232 ^ns^	-0.356 ^ns^	0.877**	0.963**	0.980**	-0.943**	-0.931**	-0.048 ^ns^	0.683 *
Heneicosane	-0.494^ns^	0.024 ^ns^	0.384 ^ns^	-0.190 ^ns^	0.943**	0.904**	0.932**	-0.872**	-0.980**	0.124 ^ns^	0.124 ^ns^
Eugenol	0.834**	0.435 ^ns^	-0.761*	-0.332 ^ns^	-0.969**	-0.566 ^ns^	-0.620 ^ns^	0.505 ^ns^	0.946**	-0.609 ^ns^	-0.049 ^ns^
Neral	0.833**	0.435 ^ns^	-0.761*	-0.331 ^ns^	-0.969**	-0.568 ^ns^	-0.621 ^ns^	0.506 ^ns^	0.947**	-0.608 ^ns^	-0.051 ^ns^
Phenylethyl Alcohol	0.193 ^ns^	-0.327 ^ns^	-0.075	0.510 ^ns^	-0.786*	-0.994**	-1.000**	0.986**	0.855**	0.217 ^ns^	-0.797 *
Methyleugenol	-0.901 **	-0.871**	0.918**	0.908**	0.521 ^ns^	-0.196 ^ns^	-0.132 ^ns^	0.268 ^ns^	-0.426^ns^	0.993 **	-0.682 *
trans-Rose oxide	-0.785*	-0.355 ^ns^	0.704*	0.236 ^ns^	0.985**	0.646 ^ns^	0.696*	-0.589 ^ns^	-0.974**	0.526 ^ns^	0.150 ^ns^
Linalool	0.905**	0.577 ^ns^	-0.851**	-0.500 ^ns^	-0.913**	-0.405 ^ns^	-0.465 ^ns^	0.337 ^ns^	0.871**	-0.744*	0.135 ^ns^
cis-Farnesol	0.897**	0.567 ^ns^	-0.842**	-0.500 ^ns^	-0.915**	-0.407 ^ns^	-0.465 ^ns^	0.337 ^ns^	0.870**	-0.744*	0.135 ^ns^
trans-Farnesol	0.904**	0.576 ^ns^	-0.850**	-0.500 ^ns^	-0.913**	-0.406 ^ns^	-0.465 ^ns^	0.337 ^ns^	0.871**	-0.744*	
trans,trans-Farnesol	0.909**	0.584 ^ns^	-0.856**	-0.500 ^ns^	-0.914**	-0.405 ^ns^	-0.465 ^ns^	0.337 ^ns^	0.870**	-0.744*	0.135 ^ns^
Yield	-0.930**	-0.784*	0.920**	0.794*	0.695*	0.024 ^ns^	0.860 ^ns^	0. 530 ^ns^	-0.611 ^ns^	0.942**	0.507 ^ns^

The different letters indicate a significant difference based on Duncan’s multiple range test at the 1% level.

**Table 6:** Comparison of the means of soil characteristics of crop sites.

Soil characteristics	Site	Mean (%) ± SD	F
Silt %	Sefidshahr	0.000 ^b^ ± 12.000	26.440**
	Yazdel	2.887 ^a^ ±18.67	
	Noushabad	0.000 ^b^ ±9.000	
Clay %	Sefidshahr	^a^ 0.000 ± 11.200	13.690**
	Yazdel	^a^ 1.155± 11.667	
	Noushabad	0.000 ^b^ ± 9.000	
Sand %	Sefidshahr	^b^ 0.000 ± 76.800	21.126**
	Yazdel	4.041 ^c^ ±69.667	
	Noushabad	^a^ 0.000 ±82.000	
Gypsum	Sefidshahr	0.000 ^b^ ±0.000	44521.000**
	Yazdel	0.000 ^b^ ±0.000	
	Noushabad	0.005 ^a^ ±0.703	
Nitrogen %	Sefidshahr	^a^ 0.002 ±0.184	784.500**
	Yazdel	^b^ 0.010 ±0.030	
	Noushabad	^c^ 0.000 ±0.010	
Organic carbon %	Sefidshahr	^a^ 0.020 ±1.848	22690.830**
	Yazdel	^b^ 0.000 ±0.150	
	Noushabad	^c^ 0.000 ±0.700	
Potassium mg/kg	Sefidshahr	^c^ 1.155 ±136.667	7639.000**
	Yazdel	^b^ 0.000 ±188.000	
	Noushabad	^a^ 0.000 ±200.000	
Phosphorus (mg/kg)	Sefidshahr	^c^ 0.123 ±0.213	26031.837**
	Yazdel	^a^ 0.000 ±13.440	
	Noushabad	^b^ 0.000 ±6.720	
Electrical conductivity (ds/m)	Sefidshahr	^b^ 0.006 ±2.737	325709.871**
	Yazdel	^c^ 0.000 ±1.769	
	Noushabad	^a^ 0.006 ±4.817	
pH	Sefidshahr	^a^ 0.003 ±7.234	5155.441**
	Yazdel	^b^ 0.006 ±7.073	
	Noushabad	^c^ 0.006 ±6.813	
Lime %	Sefidshahr	^a^ 0.006 ±23.153	179.078**
	Yazdel	^c^ 1.494 ±9.927	
	Noushabad	^b^ 0.000 ±17.950	

Assessment of irrigation water characteristics showed higher concentrations of all cations and anions, water-soluble salts and consequently higher EC, sodium adsorption ratio (SAR) and soluble sodium percentage (SSP) at the Noushabad site, which could be among the reasons for its higher EO yield. However, [61–63] showed that higher soil salinity significantly reduced EO yield in *Cuminum cyminum* L., *Mentha canadensis* L., and *Melissa officinalis* L., respectively. These conflicting results regarding the effect of salinity on EO yield of various species in previous research might be due to genotype differences (sensitivity to salinity and halophilism), growing conditions and cultivation techniques [64, 65]. Salinity may play an indirect role in EO accumulation via affecting net assimilation and/or through influencing allocation of assimilates [66]. Therefore, higher EO yield in plants under soil or water salinity conditions might be due to the reduction in primary metabolites under salinity stress that leads to use of intermediate compounds to synthesize secondary metabolites [67]. Comparison of water characteristics at Yazdel and Noushabad sites showed the lowest and highest concentrations of Mg^2+^ were recorded in Yazdel (3.82 meq/L) and Noushabad (11.04 meq/L), which might have been one of the factors influencing EO yield. Also [68] reported that, in some cases, plant vegetative growth decreased considerably in soils being irrigated with water having high magnesium concentrations, even in cases where there were no limitations on water infiltration. High magnesium concentrations in irrigation water may cause production of secondary metabolites to start thereby increasing EO yield [69].

### 3.2. Chemical composition of the EOs

The chemical analysis of the EOs identified 52 compounds that constituted 99.58–97.99% of them at the three crop sites (Table 7). The lowest number of compounds (27) was that of the EO_S_, which was significantly different from that recorded for EO_Y_ and EO_N_ (39 compounds). [38] also reported different numbers of compounds derived from Damask rose EO in various regions in Iran. Differences in the types and numbers of compounds might result from genetic variations or from environmental factors including soil chemical composition [70, 71]. Soil chemical composition affects not only the amounts of EOs but also the distribution of the chemotypes and the percentages of the compounds found in EOs because chemical elements enter into the composition of the enzymes that are involved in plant biochemical processes [45, 50]. Nonterpenoids (others) and oxygenated monoterpenes were the major constituents of the EOs at all the studied sites. These results are consistent with those of the study by [56]. The highest percentages of nonterpenoids (39.27%) and oxygenated monoterpenes (68.15%) were obtained from EO_S_ and EO_Y_, respectively.

**Table 7:** Diversity in chemical composition of *R*. *damascena* EO at the crop sites.

no.	chemical composition	RI	EO_S_	EO_Y_	EO_N_
1	α-Pinene	881.77	0.23±0.00^b^	0.32±0.00^a^	0.33±0.01^a^
2	β–Pinene	920.86	-	-	0.10±0.00^a^
3	Linalool	1026.98	-	1.25±0.00^a^	-
4	trans-Rose oxide	1027.24	1.30±0.00^a^	-	0.92±0.02^a^
5	Phenylethyl Alcohol	1054.49	-	0.98±0.01^b^	1.00±0.00^a^
6	3-Cyclohexen-1-ol, 4-methyl-1-(1-methylethyl)-, (R)-	1079.36	-	0.47±0.00^a^	-
7	L-α-Terpineol	1099.20	-	0.33±0.00^a^	-
8	Citronellol	1123.07	16.31±0.01^c^	29.05±0.00^a^	28.07±0.02^b^
9	Geraniol	1137.74	1.03±0.00^c^	6.85±0.00^a^	1.59±0.00^b^
10	Neral	1143.92	-	1.32±0.01^a^	0.25±0.01^b^
11	trans-Geranic acid methyl ester	1174.27	-	-	0.14±0.00^a^
12	2,6-Octadiene, 2,6-dimethyl-	1188.46	0. 62±0.01^b^	0.58±0.02^c^	0.80±0.00^a^
13	Eugenol	1112.13	0.85±0.01^c^	2.40±0.01^a^	1.15±0.01^b^
14	β-Elemene	1216.11	0.58±0.00^a^	-	0.33±0.01^b^
15	Geranic acid	1226.30	-	-	0.46±0.02^a^
16	Methyleugenol	1231.27	1.62±0.02^b^	0.85±0.01^c^	2.68±0.01^a^
17	Caryophyllene	1233.41	1.53±0.00^a^	1.24±0.02^a^	-
18	α-Guaiene	1242.41	1.27±0.00^b^	0.77±0.01^b^	0.65±0.00^c^
19	α-Humulene	1255.68	0.91±0.01^a^	0.62±0.02^b^	0.44±0.01^c^
20	Germacrene D	1272.03	2.37±0.00^a^	1.57±0.01^b^	0.65±0.02^c^
21	δ-Guaiene	1283.64	1.56±0.00^a^	0.87±0.00^b^	0.78±0.00^c^
22	E-Nerolidol	1317.19	-	0.23±0.00^a^	-
23	Hexadecane	1328.32	0.18±0.00^a^	0.16±0.01^b^	0.19±0.00^a^
24	8-Heptadecene	1371.67	0.48±0.01^b^	0.36±0.00^c^	0.54±0.00^a^
25	τ-Muurolol	1376.27	-	0.36±0.01^a^	-
26	Aromandendrene	1377.96	-	-	0.22±0.00^a^
27	Heptadecane	1385.23	3.10±0.01^b^	2.44±0.00^c^	3.34±0.00^b^
28	cis-Farnesol	1392.49	-	0.31±0.00^a^	-
29	trans-Farnesol	1406.29	-	2.01±0.00^a^	0.31±0.01^b^
30	trans,trans-Farnesol	1414.86	-	0.28±0.01^a^	-
31	3-Octadecene, (E)-	1425.18	-	-	0.14±0.00^a^
32	Octadecane	1436.52	0.48±0.00^a^	0.36±0.00^c^	0.45±0.00^b^
33	Benzyl Benzoate	1332.56	-	0.53±0.01^a^	0.21±0.00^a^
34	1-Nonadecene	1479.84	7.07±0.00^a^	4.45±0.00^c^	6.79±0.00^b^
35	Nonadecane	1497.84	29.66±0.00^a^	18.48±0.00^c^	20.38±0.00^b^
36	Benzene, (1-methyldodecyl)-	1498.74	-	0.41±0.00^a^	-
37	3-Eicosene, (E)-	1527.89	0.46±0.00^a^	0.22±0.00^c^	0.36±0.01^b^
38	Eicosane	1542.10	4.67±0.01^a^	2.97±0.00^b^	2.70±0.00^c^
39	Hexadecanoic acid	1547.36	-	-	1.14±0.02^a^
40	Henicos-1-ene	1577.89	0.55±0.00^b^	0.40±0.00^c^	0.57±0.01^a^
41	Heneicosane	1596.31	16.89±0.02^a^	9.99±0.01^c^	12.29±0.02^b^
42	3,7-Dimethyloct-6-en-1-yl decanoate	1601.66	-	-	0.94+0.37
43	Phthalic acid, 4,4-dimethylpent-2-yl octyl ester	1604.43	-	0.35±0.00^a^	-
44	Linoleic acid ethyl ester	1622.71	-	0.16±0.00^a^	0.17±0.00^a^
45	Linolenic acid, ethyl ester	1627.14	-	0.43±0.00^b^	0.60±0.00^a^
46	Linoleic acid	1631.85	-	0.49±0.00^a^	-
47	1-Eicosene	1634.62	0.58±0.01^a^	-	-
48	Linolenic acid	1636.84	-	-	0.90±0.00^a^
49	Docosane	1639.61	0.70±0.01^c^	0.88±0.02^b^	0.96±0.00^a^
50	Phenethyl stearate	1680.60	-	-	0.19±0.00^a^
51	9-Tricosene, (Z)-	1685.04	0.61±0.00^c^	0.92±0.00^a^	0.82±0.00^b^
52	Succinic acid, di(3,7-dimethyloct-6-en-1-yl) ester	1697.50	0.29±0.00^a^	-	-
	Total		98.45	99.58	97.99
	Monoterpenes hydrocarbons		0.85	0.9	1.13
	Oxygenated monoterpenes		18.64	39.27	30.82
	Sesquiterpenes hydrocarbons		6.66	5.07	2.42
	Oxygenated sesquiterpenes		0	3.19	0.31
	Others		68.15	51.15	63.31

^#^Retention indices (RIs) relative to n-alkanes (C6–C40) on the same methyl silicone capillary column. Values with different letters are statistically different (Duncan, p≤0.01).

ANOVA results showed that there were significant differences in the mean percentages of the compounds in *R*. *damascena* EO obtained from the studied sites (P≤ 0.01) (Table 7). [72] also reported differences in the relative percentages of the compounds in *R*. *damascena* EO obtained from different farms that they attributed to variations in the soils of a region that led to different biosynthesis and accumulation levels of volatile compounds. Therefore, identification of suitable conditions for the synthesis of metabolites and special compounds in plants can be effective in increasing their production [73]. The major constituents of the EOs at the studied sites were citronellol, nonadecane, heneicosane and geraniol with different percentages. These results are consistent with the findings of [74].

Citronellol and geraniol are the most important constituents of Damask rose EO and the main components responsible for its aroma quality [75]. The results showed the significant effect of crop site on citronellol percentage at the 1% significance level (Table 3). [54] also showed that crop site had no significant effect (p< 0.05) on carvacrol content in the species *Satureja rechingeri* Jamzad and *Satureja khuzestanica* Jamzad. These results are not consistent with those of the present study. The highest percentages of citronellol and geraniol belonged to the Yazdel site (~29.05% and ~6.85%). The differences in the percentages of these compounds at the sites are consistent with the results of the study by [26] in Iran but not with those in the studies by [74] in the western Himalayas (citronellol = 42.0% and geraniol = 21.4%) and [76] in Turkey (citronellol = 35.23% and geraniol = 22.19). Results related to correlation of soil characteristics with citronellol content showed that there were direct correlations between the amount of citronellol and soil potassium and phosphorus contents and inverse correlations between the quantity of citronellol and soil acidity and soil lime, nitrogen and organic carbon contents. Potassium plays a role in almost all plant metabolic processes the most important of which are its effects on growth, enzyme activation, and prevention of energy loss [77]. Potassium seems to be involved in the chemical structure and activation of the enzymes affecting biochemical pathways related to synthesis of active plant ingredients [50, 78]. Phosphorus is one of the macro nutrients and significantly contributes to plant growth and EO biosynthesis. It is involved in photosynthesis, respiration and pyruvate production (a necessary compound for EO biosynthesis), and is present in the structures of the three coenzymes adenosine triphosphate (ATP), coenzyme A and nicotinamide adenine dinucleotide phosphate (NADP) that take part in biosynthesis of terpenoids [79]. Soil phosphorus available to plants may be converted to calcium phosphate (unavailable to plants) under the influence of calcium carbonate in calcareous and alkaline soils of arid and semi-arid regions [78]. Therefore, reducing soil lime content can substantially help phosphorous absorption by plants. The Yazdel site had the lowest lime content. There was a direct correlation between geraniol content and soil phosphorous and silt percentage and inverse correlations between geraniol content and soil EC and soil lime and sand percentage (Table 5). Therefore, in addition to the effects of higher phosphorus and lower lime contents on the synthesis of citronellol and geraniol, lower salinity and clay texture could be among the factors that increased geraniol synthesis compared to the other sites. Also [80] showed that increased contents of the dominant compounds such as 1,8-cineole and camphor in the EO of *Achillea millefolium* L. subsp. *millefolium* were correlated with lower soil EC and higher clay percentage. Production of plant secondary metabolites is substantially influenced by environmental conditions, especially biotic and abiotic stresses. Among them, salinity greatly influences plant EO composition and their biosynthesis [81]. Production of monoterpenes is catalyzed by terpene synthesis, which is guided by stress-related programs. [62, 63, 65] determined the effects of higher salinity on the synthesis of the major compounds in the EO, especially monoterpenes, in *Mentha canadensis* L., *Melissa officinalis* L., and *Mentha spicata*, respectively. Their results contradict the findings of the present study. Higher concentrations of bicarbonate in the irrigation water at the Yazdel site and hence the greater inhibitory effect on primary metabolic activities [82], which leads to synthesis of secondary metabolites, could be another possible factor increasing the contents of citronellol and geraniol.

The citronellol-geraniol (C/G) ratio has been used in many studies to assess the aroma quality of Damask rose EO [83], which is dependent on the climatic and geographical origins of the plant [84]. The best aroma is produced when the C/G ratio is between 1.25 and 1.30 [85]. The C/G ratio varied at the studied sites (CG<17.65 in Noushabad, CG<15.85 in Sefidshahr and CG<4.24 in Yazdel). These values indicate the high-quality aroma of EO_N_. [55] showed that differences in C/G ratio in Damask rose EO were caused by harvest time. Therefore, various factors such as soil, water and even harvest time can influence the C/G ratio.

Nonadecane and heneicosane alkanes were among the most abundant compounds in Damask rose EO at all three studied sites. ANOVA results showed the significant effect of crop site on nonadecane and heneicosane quantities (P≤ 0.01) (Table 3). The highest and lowest percentages of these two compounds were obtained at Sefidshahr (29.66% and 16.89%) and Yazdel (18.48 and 9.99%), respectively. [86] in southern Iran, [74] in the Western Himalayas, and [87] in China also recorded these two compounds as the major constituents of Damask rose EO. Correlations of these two compounds with soil characteristics were assessed. The results showed that there were strong and indirect correlations between the contents of these two compounds and soil phosphorous and potassium contents and strong positive correlations between their contents and soil nitrogen, lime and organic carbon contents. Soil acidity had a significant direct correlation with the quantity of nonadecane at the 5% significance level (Table 5). High organic carbon content of the soil leads to desirable water retention that enhances root growth, and the gradual release of nitrogen increases its uptake. Nitrogen increases the growing period, plant dry materials, and photosynthesis, prepares the carbon skeleton and substrate necessary for biosynthesis of secondary metabolites and hence increases the materials that form them [88, 89]. Comparison of the correlations between nonadecane and citronellol and soil characteristics showed that the same factors increasing citronellol synthesis reduced nonadecane synthesis. Moreover, soil characteristics, except for acidity, inversely affected the amounts of heneicosane and citronellol. Therefore, crop sites with suitable soil and water characteristics for the synthesis of these alkanes lack the appropriate conditions for the synthesis of the important compounds citronellol and geraniol. In fact, plants that are grown in soils with higher lime, nitrogen and organic carbon contents and lower phosphorus and potassium contents and are irrigated with water containing lower bicarbonate levels produce EO having poorer EO aroma quality and lower amounts of alkanes.

Eugenol and neral are important compounds in Damask rose EO. ANOVA results showed that crop site had a significant effect on the quantities of these two compounds (P≤ 0.01) (Table 3), and their highest amounts (1.32–2.40%) were recorded at the Yazdel site. Previous studies did not report presence of eugenol in the chemical composition of Damask rose EO in northern Iran, but [56] found a very low amount of eugenol (0.18%) in Damask rose EO extracted in that region. These results are not consistent with those of the present research, and neral was not found in the EO_S_. [74] in the Western Himalayas, [38] in some regions of Iran, and [86] in southern Iran did not find this compound in the chemical composition of Damask rose EO either. The highest content of this compound (9.6%) has been recorded in India) [90]. Correlations between soil characteristics and the quantities of eugenol and neral were studied. The results indicated that there was a strong and positive correlation between soil phosphorus and silt percentage and the contents of these two compounds and a negative correlation between soil lime content and sand percentage and the amounts of eugenol and neral. It seems that, except for EC, the same factors affect the synthesis of these two compounds and geraniol. The trend of changes in the amounts of eugenol and neral was similar to that of geraniol. It was mainly the Sefidshahr site that did not possess the required conditions for the synthesis of these compounds.

Phenylethyl alcohol is responsible for aroma of flowers in the Rosaceae family. It is one of the major constituents of Damask rose EO [91]. It is highly soluble in water and completely vaporizes in the distillation process. Low contents of this compound are found in rose EO [92]. There were significant differences between the studied sites in the content of this material. The highest content (1.50%) was recorded at the Noushabad site. No trace of this compound was found at the Sefidshahr site. [86] in some regions of Iran and [86] in southern Iran did not find this compound in Damask rose EO either. The highest content of this compound (0.86%) was found in northern Iran and Bulgaria (27.75%). These results are not consistent with those of the present research [56, 92]. Correlations of soil characteristics with this compound were studied. The results indicated increased synthesis of this compound with decreases in soil pH and nitrogen, organic carbon, lime, potassium, phosphorus contents and also with increases in soil potassium and phosphorous contents. These results are similar to those concerning correlations between citronellol content and soil characteristics. Synthesis of the compounds responsible for the aroma of *R*. *damascena* EO seems to strongly depend on the balance between the three macronutrients (NPK) and on soil lime content. *R*. *damascena* produces high-quality EO in soils with high phosphorus, potassium, nitrogen and lime contents. [93] showed that potassium and phosphorus increased the synthesis of α-bisabolene in *Satureja hortensis* EO. However, [94] reported that potassium and phosphorus reduced synthesis of caryophyllene oxide in *Ocimum basilicum* EO. The trend of changes in cations and anions (Ca^+2^, Na^+^, CL^-^ and SO_4_^-2^), pH, EC, TDS, T.H, SSP and SAR in irrigation water corresponded to synthesis of this compound at the studied sites. Higher salinity and alkalinity of water seem to stimulate synthesis of this compound. [95] confirmed the effect of water salinity on synthesis of some compounds of EO derived from *Ocimum basilicum* cv. Keshkeni luvelou.

Methyl eugenol (ME) is another important compound in Damask rose EO that the crop site significantly influenced (P≤ 0.01) (Table 3). It is a natural carcinogenic phenylpropanoid compound [72]. Its concentration is carefully scrutinized when selecting the best option in planting Damask rose. The highest and lowest ME concentrations were found at the Noushabad (2.68%) and Yazdel (0.85%) sites. Results of correlations between soil characteristics and ME contents suggested that there were strong positive correlations between ME content and soil EC and sand and gypsum contents and strong inverse correlations between its content and soil pH and silt and clay percentages. [96] confirmed that EC increased the contents of important compounds in the EO of *Melissa officinalis* L. These results are consistent with those of the present research. Higher EC values pose major constraints to growth, development, productivity, and crop quality in many regions of the world through disrupting plant physiological functions, but they may stimulate production and accumulation of secondary metabolites in plants [61]. High levels of Mg^+2^ in irrigation water may also increase synthesis of ME.

Linalool and farnesol have important therapeutic effects, especially antimicrobial activity. Low contents of linalool and farnesol isomers in EO_Y_ are among the other advantages of this EO that are consistent with the findings of [97]. There was a strong direct correlation between silt percentage and phosphorus content and the contents of linalool and farnesol isomers (cis-farnesol, trans-farnesol and trans,trans-farnesol). Strong indirect correlations were also found between the contents of these materials and soil EC, lime content and sand percentage. These results completely match the correlations between geraniol and soil characteristics (Table 5). Therefore, heavy-textured saline soils with high lime and phosphorous contents will substantially help synthesis of this type of compounds in Damask rose EO. [45] found a negative correlation between sand percentage and linalool content and a positive correlation between soil phosphorous content and α-terpinyl acetate in *Thymus pulegioides* EO.

Rose oxide is also one of the effective compounds in the flavor of Damask rose EO. Even low amounts of this compound have a substantial effect on aroma and flavor of this EO. Trans-rose oxide quantities varied at the studied sites. Its highest amount (1.30%) was recorded at the Sefidshahr site, but no trace of it was found at the Yazdel site. [56] reported only low contents of rose oxide (0.1%) from northern Iran. These results were not consistent with those of the present research. There were direct correlations between rose oxide content and soil lime and organic contents and sand percentage and inverse correlations between its quantity and soil phosphorous content and silt percentage. It seems that it is necessary to increase the factors reducing the contents of the other compounds responsible for the aroma of Damask rose EO in order to increase the quantity of rose oxide in it. This will considerably influence the low quality of Damask rose EO.

### 3.3. Antimicrobial and antifungal activities of essential oil

**Fig 1:**
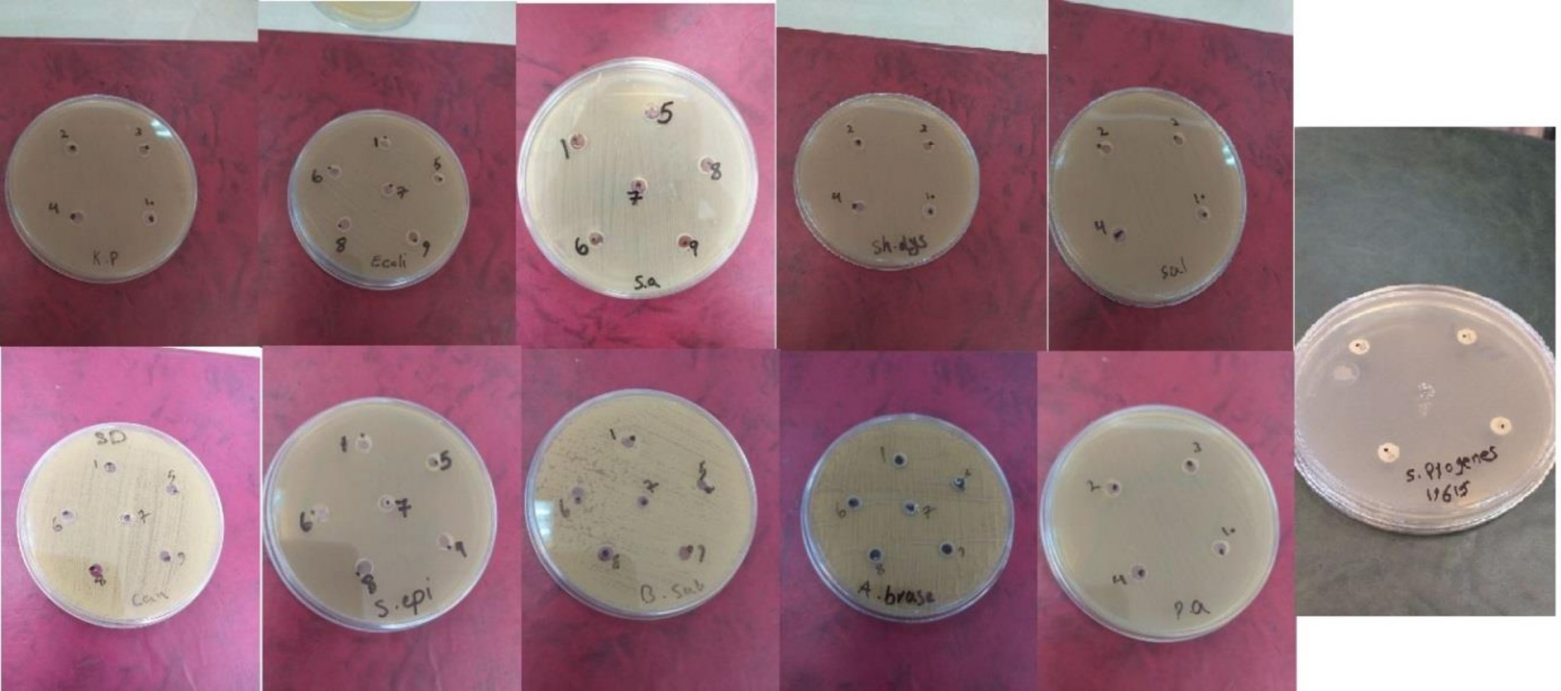
Agar well-diffusion of *R*. *damascene* EOs.

Antifungal and antibacterial activities of Damask rose EO against different strains were assessed using the agar well-diffusion method (Table 8 and Fig 1). Inhibition zones (IZs) were only observed for Gram-positive *Streptococcus pyogenes* and *Aspergillus brasiliensis*. ANOVA results showed that crop site had a significant effect on IZD of Damask rose EO against *Streptococcus pyogenes* and *Aspergillus brasiliensis* (P≤ 0.01) (Table 9). [98] assessed the effect of crop site on IZs of *Tagetes minuta* L. EO against different microbial strains using the agar well-diffusion method. The largest IZD of rose EO against *Aspergillus brasiliensis* (~14.67 mm) belonged to the Sefidshahr site, that exhibited relatively potent antifungal activity (~30 mm) compared to nystatin. These results are consistent with those of the study by [99] who determined antifungal activity of Damask rose EO extracted in Isfahan against *Aspergillus brasiliensis*. Variations in antimicrobial activities of EOs of a species grown in different regions may result from differences in the dominant compounds found in the Eos and the presence of different chemicals in the EOs [98, 100]. Dominance of alkanes (especially nonadecane and heneicosane) in EO_S_ can be one of the reasons for antifungal activity. Not only dominant and main compounds in EOs but also the synergistic effects of those compounds with lower percentages may contribute to their antimicrobial activity [101]. Therefore, the low contents of trans-rose oxide and sesquiterpenes (e.g., germacrene D, caryophyllene, α-guaiene, α-humulene and δ-guaiene) in EO_S_, which were higher compared to the other two sites, can probably be among the other factors influencing this antifungal activity. The effects of abundance of sesquiterpenes against different microbial strains were recorded by [102] who showed that antifungal activity of Eos obtained from Phlomis species. It is noteworthy that the value of MBC and MIC obtained for this fungus was 1000 μg/mL, which is much higher than that of nystatin (31.2 μg/mL) (Table 10).

**Table 8:** IZDs of *R*. *damascene* EO at the crop sites and of the antibiotics against the standard microbial strains.

microbial strains	IZDs (mm)					
	EOs			Antibiotics		
	Sefidshahr	Yazdel	Nooshabad	Rifampin	Gentamicin	Nystatin
*S*. *aureus*	ND	ND	ND	21	27	NA
*S*. *epidermidis*	ND	ND	ND	27	45	NA
*B*. *subtilis*	ND	ND	ND	19	30	NA
*Sh*. *dysenteriae*	ND	ND	ND	9	17	NA
*P*. *aeruginosa*	ND	ND	ND	ND	20	NA
*E*. *coli*	ND	ND	ND	10	23	NA
*K*. *pneumonia*	ND	ND	ND	8	17	NA
*S*.*pyogenes*	11.67±0.58^a^	11.67±0.58^a^	ND	21	32	NA
*S*. *paratyphi-A*	ND	ND	ND	8	18	NA
*C*. *albicans*	ND	ND	ND	NA	NA	33
*A*. *brasiliensis*	14.67±0.58^a^	ND	ND	NA	NA	30

Results are expressed as means ± SD of triplicate values. ND: not determined. NA: no activity. Values with different letters are statistically different (Duncan, p≤0.01).

**Table 9:** ANOVA related to the effect of crop site on IZD of *R*. *damascena* EO for some standard microbial strains.

Source of variation	df	MS	
		*S*. *pyogenes*	*A*. *brasiliensis*
Site	2	32.111**	75.111**
Error	6	0.222	0.111

** 1% level of probability is significant.

**Table 10:** MIC, MBC, and MFC values of *R*. *damascena* EO at the crop sites and of the antibiotics against some standard microbial strains.

Microbial strains	MIC (μg/mL)			MBC/MFC (μg/mL)			Antibiotics		
	EO_S_	EO_Y_	EO_N_	EO_S_	EO_Y_	EO_N_	Rifampin	Gentamicin	Nystatin
*S*. *aureus*	31.25	500	1000	62.50	>1000	>1000	31.25	1.95	NA
*S*. *epidermidis*	500	1000	500	>1000	>1000	>1000	1.95	1.95	NA
*B*. *subtilis*	125	500	250	>1000	>1000	>1000	31.25	3.90	NA
*Sh*. *dysenteriae*	500	500	250	1000	1000	500	15.36	3.90	NA
*P*. *aeruginosa*	31.25	31.25	62.50	1000	>1000	>1000	31.25	7.81	NA
*E*. *coli*	125	125	125	1000	>1000	>1000	15.63	31.25	NA
*K*. *pneumonia*	125	500	250	1000	1000	1000	15.63	3.90	NA
*S*.*pyogenes*	<15.63	<15.63	125	<15.63	<15.63	125	0.975	0.975	NA
*S*. *paratyphi-A*	250	500	250	1000	1000	500	15.63	3.90	NA
*C*. *albicans*	31.25	<15.63	250	31.25	<15.63	250	NA	NA	125
*A*. *brasiliensis*	1000	1000	500	1000	1000	500	NA	NA	31.2

The IZDs of EO_S_ and EO_Y_ for the Gram-positive species *Streptococcus pyogenes* was ~11.67 mm, which was relatively good compared to those of rifampin (~21 mm) and gentamicin (~32 mm). The findings also indicated that the MIC and MBC values of EO_S_ and EO_Y_ for *Streptococcus pyogenes* were <15.63 μg/mL, which were considerably higher than those of rifampin (0.975 μg/mL) and gentamicin (0.975 μg/mL). The EO_S_ and EO_Y_ also exhibited potent antibacterial activity against the Gram-negative species *Pseudomonas aeruginosa* (MIC = 31.25 μg/mL) compared to rifampin (31.25 μg/mL) and relatively good activity compared to gentamicin (7.81 μg/mL). However, EO_N_ exhibited much weaker antibacterial activity (MIC = 62.5 μg/mL). [87] showed that Damask rose EO had no antibacterial activity against *Pseudomonas aeruginosa*. These findings are consistent with the results of the present research. Similar antibacterial activities of EO_S_ and EO_Y_ seem to be due to their similar chemical profiles, especially with respect to sesquiterpene hydrocarbons. Caryophyllene was observed in EO_S_ and EO_Y_, but was not found in EO_N_. Moreover, given the same antibacterial properties of the two Eos, we can infer that the various relative contents of the compounds in their Eos were the reason for their identical antibacterial activities. Dominance of citronellol, geraniol, neral, and eugenol the unique presence of linalool and farnesol isomers in EO_Y_, can be the most effective factors responsible for its antimicrobial activity. Antimicrobial activities of these compounds against various microbial strains were confirmed in different studies [103, 104]. Conversely, dominance of alkanes (e.g., nonadecane and heneicosane, 1-nonadecene, and eicosane) and higher quantities of sesquiterpenes in Eos can be effective factors responsible for the antimicrobial activity of this EO. Many studies have confirmed the strong antimicrobial effects of alkanes and sesquiterpenes [50, 103, 105]. Diversity in chemical composition of EOs lead to different main activities in relation to possible antimicrobial activities of plant EOs including disruption of the cytoplasmic membrane that disrupt the driving force of protons, electron flow, active transport, and also coagulation of cell contents [106]. Although Musk rose EO from all the studied sites did not create effective inhibition zones against the bacterial and yeast strains, their various concentrations were effective in inhibiting and killing them. Their MBC and MIC values against some strains were considerable compared to those of the studied antibiotics. EO_Y_ exhibited the strongest inhibitory and lethal activity against *Candida albicans* (MIC and MBC <15.63 μg/mL), very considerable and significant compared to those for nystatin (125 μg / mL). It was six times more effective than nystatin. These findings are not consistent with the findings of [24] in Bulgaria. Although the MIC and MBC values for EO_S_ (62.5 μg/mL) and EO_N_ (250 μg/mL) against *C*. *albicans* were higher than those of EO_Y_, they were found to be more potent than nystatin. The high potency of EO_Y_ against *C*. *albicans* is probably due to the presence of citronellol, geraniol, neral, eugenol, linalool, and farnesol isomers compared to EO_S_ and EO_N_. Citronellol [107], geraniol [108] and farnesol [109] were found to be effective against *Candida* species. Lower potency of EO_N_ compared to EO_Y_ against *C*. *albicans* can be due to lower quantities or the absence of these compounds. However, the low amounts of these compounds in EO_S_ were probably compensated for by the high quantities of alkanes and sesquiterpenes in this EO, which enabled EO_S_ to inhibit and kill *C*. *albicans* at a lower MIC value compared to EO_N_.

Another considerable activity of the Damask rose EOs was the antibacterial activity of EO_S_ against the Gram-positive *Staphylococcus aureus* (MIC = 31.25 μg/mL and MBC = 62.5 μg/mL). It was as effective against this bacterial species as rifampin (MIC = 31.25 μg/mL). This finding is not consistent with those of the research by [24] in Bulgaria (MIC = 60 μg/mL) and [87] in China (MIC = 250 μg/mL) for Musk rose EO. [87] confirmed the inhibitory and lethal effects of linalool, phenylethyl alcohol, citronellol, geraniol, farnesol, and methyl eugenol derived from Musk rose EO against *Staphylococcus aureus*. Farnesol showed the lowest MIC and MBC values (MIC = 31.25 μg/mL and MBC = 62.5 μg/mL). The contents of these compounds were much lower in EO_S_ than in EO_N_ and EO_Y_. Therefore, antibacterial activity of EO_S_ against *Staphylococcus aureus* might be due to higher amounts of alkanes that compensated for lower quantities of the above-mentioned compounds whereas EO_N_ and EO_Y_ had low quantities of alkanes and hence lower antibacterial activity.

The studied EOs only exhibited identical MIC value (MIC = 125 μg/mL) against the Gram-negative species *Escherichia coli*, and were less potent than gentamicin. These findings are consistent with those of [87] regarding antibacterial activity of Musk rose EO against *E*. *coli* (MIC = 125 μg/mL) in China. Inhibitory effects of geraniol [110] and eugenol [111] against *E*. *coli* were confirmed in previous studies. Similarly, [87] attributed the inhibitory and lethal effects against *E*. *coli* to the presence of linalool, phenyl alcohol, β-citronellol, geraniol, eugenol, an methyl eugenol, with β-citronellol and geraniol showing the strongest antibacterial activity against this this bacterial species (MBC = 125 μg/mL and MIC = 62.5 μg/mL). Therefore, all the above compounds derived from the studied EOs, except for phenylethyl alcohol, were present at all three sites in different quantities and could be responsible for the antibacterial activity of the EOs.

EO_S_ at low amounts exhibited antibacterial activity against the other studied strains including those of the Gram-positive *Staphylococcus epidermidis* and *Bacillus subtilis* and the Gram-negative *Klebsiella pneumoniae* and *Salmonella paratyphi*-A serotype, showing the remarkable effects of its content of alkanes in this activity. MIC values of EO_S_ were lower than EO_N_ and EO_Y_. EO_N_ was less potent than EO_S_ and EO_Y_. The MIC and MBC values of EO_N_ were only lower than those of EO_S_ and EO_Y_ against *Sh*. *Dysenteriae* and *A*. *brasiliensis*.

In general, EO_S_ had the strongest inhibitory and lethal effects against the various studied strains, especially against the Gram-positive bacteria. EO_Y_ showed the strongest inhibitory and lethal effects against the yeast strains and effects similar to those of EO_S_ against some of the Gram-negative bacterial strains.

## 4. Conclusion

The present study showed that yield, chemical composition and antimicrobial activity of *R*. *damascena* EO were influenced by different soil and irrigation water characteristics at the crop sites. The correlations between EO yield and soil EC and gypsum and lime contents and components of soil texture revealed that *R*. *damascena* should be planted in light-textured soil profile with high EC and gypsum and lime contents and be irrigated with high-salinity cation- and anion-rich water to produce the highest yield, that Noushabad site had the most suitable conditions for production of highest yield EO (~0.0266%). However, EO_Y_ with the highest concentrations of geraniol (~29.05%), citronellol (~6.85%), eugenol (~2.40%), neral (~1.32%), and linalool (~1.25%), and having the lowest quantities of alkanes, produced the best quality rose EO with respect to aroma and flavor. High levels of phosphorus and potassium and low EC and low soil lime and nitrogen contents and sand percentage and irrigation with water containing low quantities of magnesium cations were among the most effective factors increasing the amounts of compounds responsible for the quality of the EO. The differences in the chemical composition of the EOs led to various antimicrobial activities against the different microbial strains. In general, the EO_S_ showed the strongest antimicrobial activity against the microbial strains due to its high contents of alkanes and sesquiterpenes, especially against *Staphylococcus aureus* (MIC = 31.25 μg/mL and MBC = 62.5 μg/mL) and *Pseudomonas aeruginosa* (MIC = 31.25 μg/mL). Dominance of the compounds producing high-quality flavor and aroma in the EO_Y_ also led to the strongest inhibitory and lethal effects against *Candida albicans* (MIC and MBC <15.63 μg/mL). In all, EOY is a suitable option for aroma and flavor quality, the EON for yield, and EOS for inhibiting and killing some microbial strains a potentially promising strategy.

## Supporting information

S1 Fig GC–MS chromatogram of essential oil of *R. damascena* from Sefidshar. (DOCX)

S2 Fig GC–MS chromatogram of essential oil of *R. damascena* from Yazdel. (DOCX)

S3 Fig GC–MS chromatogram of essential oil of *R. damascena* from Noushabad. (DOCX)
