# Supplementary figures and images for: Relationships of irrigation water and soil physical and chemical characteristics with yield, chemical composition and antimicrobial activity of Damask rose essential oil
Source: PLoS One. 2021 Apr 16;16(4):e0249363. doi: 10.1371/journal.pone.0249363 (PMC8051937; doi:10.1371/journal.pone.0249363)

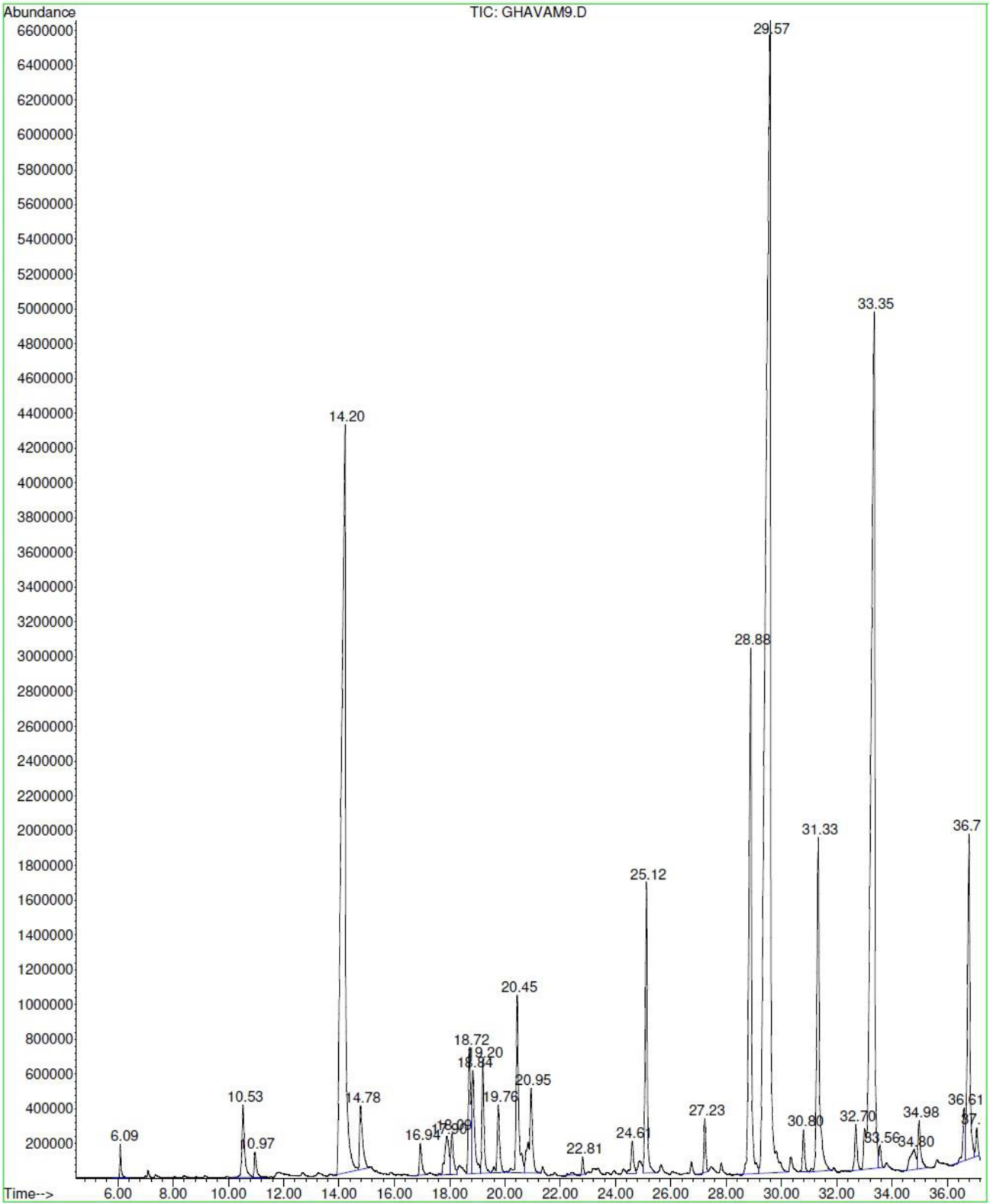


**S1 Fig.** GC–MS chromatogram of essential oil of *R. damascena* from Sefidshar

Supplement: S1 Fig — (DOCX) [file pone.0249363.s001.docx]

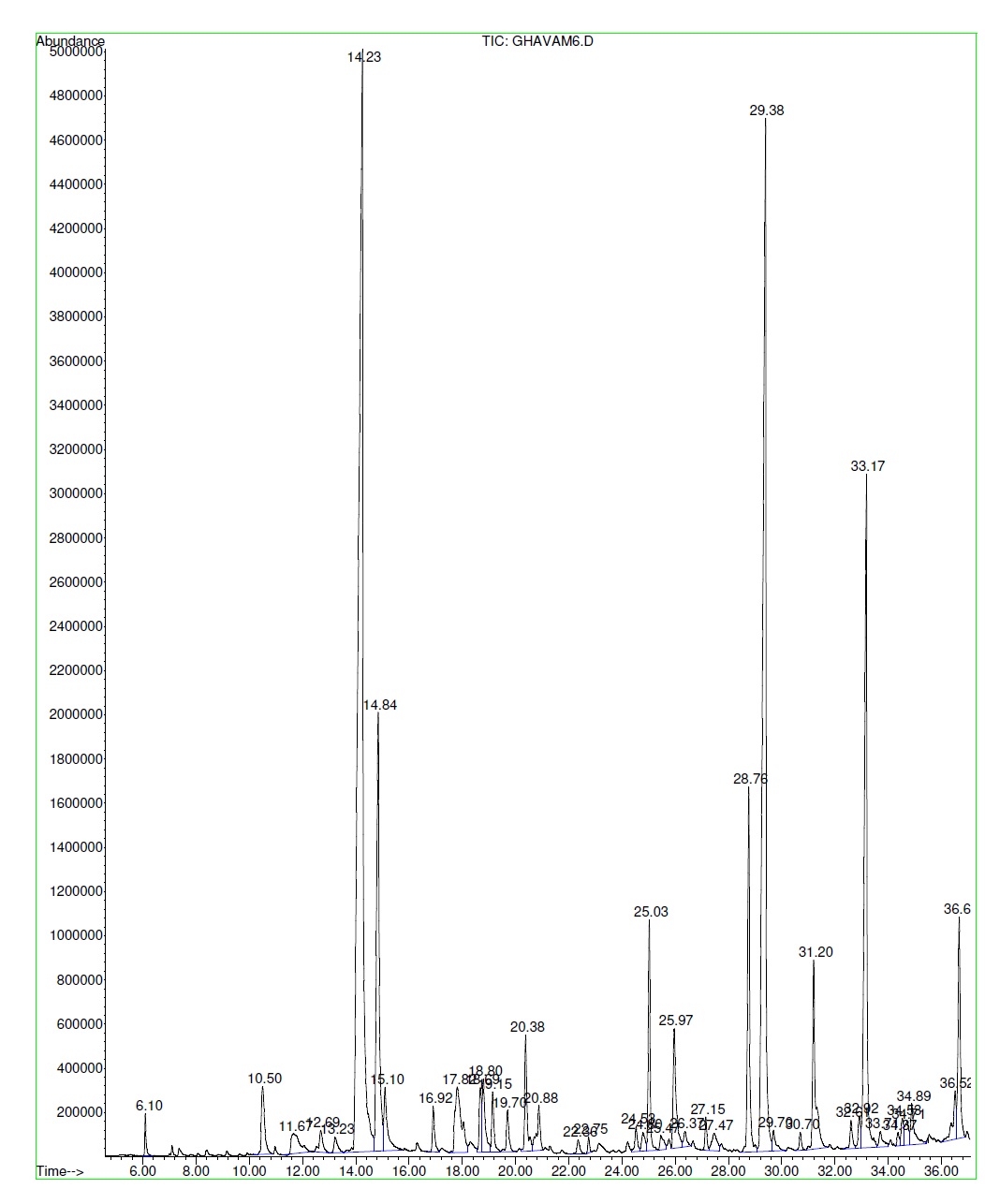


**S2 Fig.** GC–MS chromatogram of essential oil of *R. damascena* from Yazdel

Supplement: S2 Fig — (DOCX) [file pone.0249363.s002.docx]

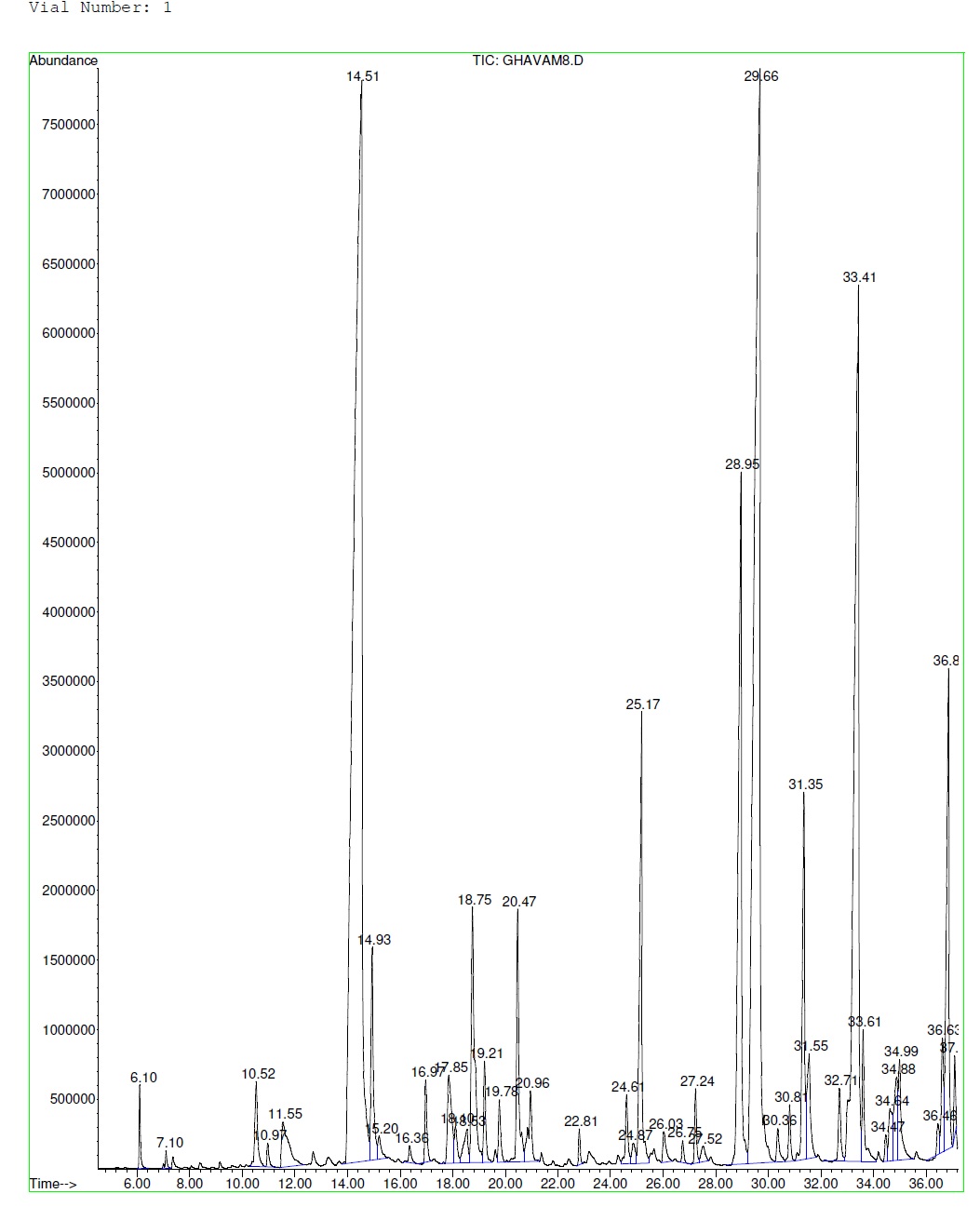


**S3 Fig.** GC–MS chromatogram of essential oil of *R. damascena* from Noushabad

Supplement: S3 Fig — (DOCX) [file pone.0249363.s003.docx]
